# The Role of CYP2E1 in the Drug Metabolism or Bioactivation in the Brain

**DOI:** 10.1155/2017/4680732

**Published:** 2017-01-10

**Authors:** W. A. García-Suástegui, L. A. Ramos-Chávez, M. Rubio-Osornio, M. Calvillo-Velasco, J. A. Atzin-Méndez, J. Guevara, D. Silva-Adaya

**Affiliations:** ^1^Departamento de Biología y Toxicología de la Reproducción, Instituto de Ciencias, Benemérita Universidad Autónoma de Puebla, 72000 Heroica Puebla de Zaragoza, PUE, Mexico; ^2^Departamento de Medicina Genómica y Toxicología Ambiental, Instituto de Investigaciones Biomédicas, Universidad Nacional Autónoma de México, 70228 Mexico City, Mexico; ^3^Laboratorio Experimental de Enfermedades Neurodegenerativas, Instituto Nacional de Neurología y Neurocirugía, 14269 Mexico City, Mexico; ^4^Departamento de Investigación en Bioquímica, Instituto Nacional de Enfermedades Respiratorias, 14080 Mexico City, Mexico; ^5^Departamento de Bioquímica, Facultad de Medicina, Universidad Nacional Autónoma de México, 04510 Mexico City, Mexico

## Abstract

Organisms have metabolic pathways that are responsible for removing toxic agents. We always associate the liver as the major organ responsible for detoxification of the body; however this process occurs in many tissues. In the same way, as in the liver, the brain expresses metabolic pathways associated with the elimination of xenobiotics. Besides the detoxifying role of CYP2E1 for compounds such as electrophilic agents, reactive oxygen species, free radical products, and the bioactivation of xenobiotics, CYP2E1 is also related in several diseases and pathophysiological conditions. In this review, we describe the presence of phase I monooxygenase CYP2E1 in regions of the brain. We also explore the conditions where protein, mRNA, and the activity of CYP2E1 are induced. Finally, we describe the relation of CYP2E1 in brain disorders, including the behavioral relations for alcohol consumption via CYP2E1 metabolism.

## 1. Introduction

The most important determinant in the persistence, bioavailability, and subsequent toxicity of a xenobiotic in the organism is the capacity to be metabolized and excreted [1]. The brain is a heterogeneous organ in which each region and cell type has a different metabolic capacity and therefore a selective cellular answer to different xenobiotics [2]. Xenobiotics are substrates of two different, general reactions through their biotransformation. In phase I reactions, a polar reactive group is introduced into the molecule. This type of reaction includes oxidations (cytochrome P450, monoamine oxidase, alcohol dehydrogenase, etc.), reductions (carbonyl reduction, sulfoxide reduction, quinone reduction, etc.), and hydrolysis (esterases, peptidases, etc.). After the polar group is aggregated, these compounds are the target for a second type of reaction: phase II reactions [1]. These conjugation enzymes include sulfotransferases, UDP glucuronosyltransferase, and glutathione S-transferase. These enzymes aggregate heavy substituents, like sugars, sulfates, or amino acids. These substituents enhance xenobiotic solubility and facilitate its elimination outside the body. Even though this biotransformation precedes detoxication, reactive intermediaries are formed and result in more harmful compounds than the original. These compounds are called bioactive compounds. This activation or bioactivation is the initial event for a lot of chemically induced toxicity [3]. The metabolism of endogenous compounds and 90% of exogenous drugs currently in use are governed by the highly polymorphic enzyme, cytochrome P450 (CYP450) [4]. Specifically, CYP2E1 has been implicated in different brain pathologies, possibly due to its role as a drug metabolism or activator enzyme.

## 2. CYP450 in the Brain

CYP450 is a group of enzymes found in microsomal protein fractions with monooxygenase activity, principally in the mitochondrion [5]. CYP450 exerts its actions by three catalytic enzymatic reactions. The first catalytic activity of CYP enzymes is the activity as a monooxygenase, activating molecular oxygen with electrons from NADPH via NADPH-CYP450 reductase and inserting one atom of molecular oxygen into the substrate, followed by a second catalytic activity, commonly referred as an oxidase activity. This involves electron transfer from reduced CYP450 to molecular oxygen with the formation of a radical superoxide anion and hydrogen peroxide. The third catalytic activity of the P450 system, known as reductase activity, involves direct electron transfer to reducible substrates such as quinones and proceeds under anaerobic conditions [6]. CYP450 phase I enzymes are involved in the oxidation or deactivation of endogen and exogenous compounds such as hormones, fatty acids, drugs, and toxins present in the environment and in the diet [7]. CYP450s are principally present in the liver, adrenal cortex, kidney, and lungs and in fewer concentrations in the brain, which represents 1% of the concentration found in the liver [8–10]. CYP450s are present in the brain in many different subcellular membrane compartments, including the plasma membrane, endoplasmic reticulum, Golgi apparatus, peroxisomes, lysosomes, and mitochondria [11–14]. Specially CYP1A1, CYP2B1, CYP2D6, and CYP2E1 have been found in significant amounts in other cell compartments, particularly within the mitochondria of different species including humans [15].

Brain regions vary in composition, density, and cellular type. As expected, the CYP450 brain localization is heterogeneous and its levels vary in different brain regions for the distinct 1, 2, and 3 subfamilies [16]. The most relevant enzymes present in the brain are CYP1B1, CYP2D6, CYP2E1, CYP2J2, CYP2U1, and CYP46A1 [17], with heterogeneous distributions in different brain areas. The dura mater has a different composition from other brain structures, with high levels of CYP1B1 and a lesser expression of CYP1A1, AYP2U1, CYP3A5, CYP2R1, CYP2D6, and CYP46A1 [17]. CYP450 participates in the metabolism of different compounds in the brain such as drugs; antidepressants; antipsychotics; neurotoxins, like ethanol, nicotine, organophosphorus pesticides, and so forth; and endogen compounds, like fatty acids, steroids, and neurotransmitters. CYP450 families 1A, B, 2B, C, D, E, and 3A participate in the xenobiotic brain transformation [16, 18]. In humans, the expression of different types of CYP450 in the brain is as follows: CYP1A1 in mesencephalon, cortical structures, basal ganglia, and cerebellum [19]; CYP3A5 is expressed in the pituitary gland [20]; CYP1B1 is localized in the cell nucleus or in the temporal lobule and putamen [19, 21]; CYP2B6 is expressed in high levels in the cerebellum, basal ganglia, and at lesser levels in cortical regions and the hippocampus. CYP2C13 is expressed in a homogenous way in regions such as the basal ganglia, cortex, hippocampus, and the olfactory bulb. The presence of CYP2D6 is high in the cerebellum, the hippocampus, and the cortex. The European Bioinformatics Institute Expression Atlas indicates, in general, that CYP450 expression is higher in the liver than in the brain. The expression of CYP46A1, which is highly expressed in the brain and has null expression in the liver, is interesting. CYP1B1 and CYP2U1 are expressed twice as much in the brain than in the liver. The expression of CYP1A, CYP2C8, CYP2C19, CYP3A4, CYP3A5, and CYP2E1 seems to be negligible in the human brain [22].

CYP450 has been principally reported in some neurons and sometimes in glial cells [7]. CYP1B1 has been identified in microvessels and has an emphasized role of quantitative importance in the brain blood barrier (BBB) [23]. CYP1B1 and CYP2U1 transcripts were mainly detected in brain microvessels, whereas no other CYP proteins were detected [22]. BBB expressed different CYP450 isoforms in high levels, forming a metabolic barrier, regulating the blood flow, compound flow, and signal during inflammation. In neurons, CYP450 has other functions: in regions, such as the hypothalamus, the hippocampus, and the striatum, it provides signaling molecules (steroids and fatty acids) for maintenance of neuronal extension [24]. CYP450s are induced by the presence of xenobiotics such as nicotine, ethanol, acetone, and phenobarbital [25]. CYP2E1 is a metabolic enzyme, but its expression and activity in the Central Nervous System (CNS) is not completely understood. An altered expression has been observed in normal physiology and pathology of the CNS and other human tissues.

## 3. CYP2E1 in the Brain

CYP2E1 is an enzyme that particularly participates in the metabolism of endogenous substrates, including acetone and fatty acids (abundant in the brain) [26] and exogenous compounds such as anesthetics, ethanol, nicotine, acetaminophen, acetone, aspartame, chloroform, chlorzoxazone, tetrachloride, and some antiepileptic drugs like phenobarbital. CYP2E1 can also activate toxic compounds and procarcinogens found in tobacco smoke and nitrosamine compounds [26–33]. CYP2E1 has an important player in the microsomal ethanol oxidizing system (MEOS). After chronic ethanol consumption, the activity of the MEOS increases, with an associated rise in cytochrome P450, especially CYP2E1, and the proliferation of the smooth endoplasmic reticulum (SER) [34]. Excessive alcohol consumption induces an endoplasmic reticulum (ER) stress response, a condition under which unfolded/misfolded protein accumulates in the ER, contributing to alcoholic disorders of major organs such as the liver, pancreas, heart, and brain [35].

CYP2E1 has been founded in significant amounts in different cell compartments, including the endoplasmic reticulum, the plasma membrane, and the Golgi apparatus [36]. CYP2E1 is highly expressed in rat brain mitochondria [12]. CYP2E1 targeting to the correct subcellular compartment requires only one type of signal. Proteins targeted to the endoplasmic reticulum, the Golgi apparatus, and the plasma membrane, as well as secreted proteins, are first targeted to the endoplasmic reticulum through a SRP dependent mechanism. In contrast, mitochondrial targeting is mostly a posttranslational event which requires protein translocation to the mitochondrial matrix by the outer and inner membrane transporters [15].

Even though most of the studies have indicated that levels CYP2E1 mRNA and protein in rat brains are extremely low, both were detected in the olfactory lobe [37–40], in the neurons of the cortex, cerebellum, and hippocampus, mainly in the microsomal fraction [41]. CYP2E1 protein levels in rat brains were reported to be 25% that of liver levels [42]. Studies of rat brains describe a constitutive CYP2E1 expression in pyramidal neurons of the frontal cortex, cortical astrocytes, the polymorphic cell layer in the hippocampus, the olfactory lobe, and endothelial cells, but not in the granule cells of the dentate gyrus nor in the Purkinje cells of the cerebellum [11, 43]. In cerebral blood vessels, CYP2E1 is associated with astrocytic end-feet [41, 43, 44]. In contrast, other studies demonstrate higher CYP2E1 protein levels in the cerebellum and olfactory bulb compared to other regions of the rat brain [42, 45, 46]. The olfactory lobes exhibit the highest CYP2E1 protein expression and catalytic activity in control rats [30, 47]. CYP2E1 protein expression is founded [23, 41, 47]. Human brain mRNA CYP2E1 expression is detected in all the brain regions examined, principally in the neurons of the cortex, cerebellum, and hippocampus. The red nucleus and substantia nigra exhibit lower levels of CYP2E1 mRNA compared to other regions [41, 48]. Human CYP2E1 protein expression was detected in the brains of nonalcoholic nonsmokers in granular cells of the dentate gyrus, pyramidal cells of the hippocampus, and pyramidal neurons of the frontal cortex. CYP2E1 protein expression in the saline-treated brains of monkeys resembles the CYP2E1 distribution in the brains of nonalcoholic nonsmokers, suggesting significant differences in expression between rodent and primate brains [49]. CYP2E1 mRNA is also found in the eye, specifically in the human retinal pigment epithelium [50]. In human prenatal cephalic tissues, CYP2E1 mRNA, protein, and enzymatic activity were documented during the first and second trimesters of pregnancy [51, 52]. Glial and neuronal cell cultures exhibit a higher activity of CYP2E1 compared with the liver CYP2E1 enzyme [53].

The protein, mRNA, and catalytic CYP2E1 is detected in rodents, human, and nonhuman primates (Table 1). In all cases the presence in regions is specifically cellular dependent. These findings support the fact that the different brain's needs depend on the cell and region and tell us about the vulnerability or the efficacy metabolism in different regions to different compounds substrate of CYP2E1.

## 4. Protein, mRNA, and Activity Modulation of CYP2E1 in the Brain

A variety of heterocyclic compounds such as imidazole, pyrazole, 4-methylpyrazole, thiazole, isoniazid, solvents such as dimethyl sulfoxide, various alcohols, benzene, and acetone have been shown to elevate CYP2E1 levels [54]. In the same way as with many pathophysiological conditions, such as obesity, diabetes, fasting, and cancer, nonalcoholic steatohepatitis CYP2E1 expression is elevated [26, 30, 55–58].

**Figure 1:**
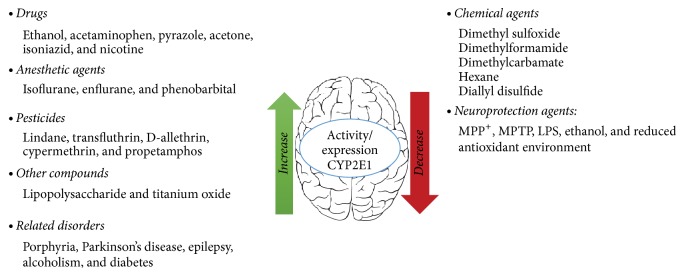
Exogenous agents and pathologies associated with CYP2E1 activity and expression in brain. LPS: lipopolysaccharide; MPP+: 1-methyl-4-phenylpyridinium; MPTP: 1-metil-4-fenil-1,2,3,6-tetrahydropyridine.

Initially observed with ethanol, a substrate of CYP2E1, many of the substrates of CYP2E1 can induce their own metabolism and elevate CYP2E1 expression. CYP2E1 induction by ethanol contributes to an increase in the ethanol metabolism observed in alcoholics (Figure 1) [28, 57], whereas the major ethanol metabolizing enzyme in the liver is alcohol dehydrogenase I (ADH) [2], which is not found in the brain [34]. Ethanol metabolism oxidation to acetaldehyde occurs in the brain [59].

Several studies have evaluated the effects of ethanol over the CYP2E1 protein, mRNA levels, and activity. In the rat brain, ethanol treatment (3.0 g/kg, 30 days) regions such as the cerebellum, hippocampus and the brainstem are cellular specific induction of CYP2E1 protein and activity by ethanol, accompanied by ethanol-induced reactive oxygen species (ROS) generation and neuronal degeneration [60]. Other studies report that ethanol treatment (3.0 g/kg, 7 days) in rats significantly increases CYP2E1 levels in the olfactory bulbs, the frontal cortex, the hippocampus, and the cerebellum [11, 61]. Rats pretreated with ethanol, pyrazole, or acetone have increased microsomal CYP2E1 brain activity. Ethanol treatment (0.8 mL/kg, one day) increases the levels of mRNA expression and activity in the cerebellum and hippocampus, and there is a relatively small increase in the olfactory lobes but no significant change in other brain regions. In the same study, in vitro assay using CYP2E1 inhibitors (dimethyl sulfoxide, dimethylformamide, hexane, and diallyl disulfide) show an inhibited N-nitrosodimethylamine (NDMA) activity that indicates that, like the liver, NDMA-activity in rat brains is catalyzed by CYP2E1 [46]. In cortical glial cultures, low concentrations of ethanol cause increased activity of CYP2E1 [40]. In the human ARPE-19 retinal epithelium pigment cell line, exposure to ethanol augmented CYP2E1 mRNA and CYP2E1 protein activity, accompanied with the formation of ROS in an alcohol dependent manner [50]. This demonstrates that chronic alcohol ingestion could enhance the sensitivity of certain regions of the brain to neurodegeneration induced by these substances or by other exogenous compounds.

Tobacco smoke has been shown to have greater ethanol metabolism rates and to induce the ethanol metabolizing enzyme CYP2E1 in animals and humans [62–65]. Nicotine treatment (1.0 mg/kg, 7 days) increases CYP2E1 levels in olfactory bulbs, the frontal cortex, cerebellum, and brainstem. This induction is cell type specific in the rat brain [11]. Chronic nicotine treatment increases CYP2E1 in the rat brain. On the other hand, chronic 7-day (1 mg/kg, 7 days, s.c.) treatment increases CYP2E1 in the frontal cortex, hippocampus, and cerebellum, returning to basal levels 24 h after the last injection. In contrast, acute nicotine treatment only induces CYP2E1 levels in the cerebellum [44]. Chronic nicotine treatment (average 0.6 mg/kg, 22 days, similar to the average daily amount received by a smoker) induces CYP2E1 expression in the pyramidal neurons in the frontal cortex and in the Purkinje cells in the cerebellum. The expression pattern in monkey brains following a chronic nicotine treatment is similar to that of smokers, suggesting that nicotine may be the primary component in cigarettes that induce CYP2E1 [44]. After ethanol treatment, the CYP2E1 protein and mRNA ethanol induction exhibit greater magnitudes in the neuronal cells than in glial cells [53], and human neuroblastoma IMR-32 cells show a higher nicotine induced CYP2E1 expression [11]. Humans exposed to nicotine, smokers, and patients receiving nicotine treatments (patients with Alzheimer's disease, Parkinson's disease, or ulcerative colitis) may have altered CYP2E1 levels and activity, mediated metabolism of drugs and toxins, and altered toxicity generated by the CYP2E1 metabolism [44].

From 80 to 95% of alcoholic smokers, compared with 25–30% of nonalcoholic smokers, consume twice as much alcohol as nonsmokers [64]. Following this line, consistent nicotine administration to rats increases their self-administration of ethanol [65]. Nicotine pretreatment of rats increases voluntary ethanol intake compared to saline pretreatment and enhanced CYP2E1 levels correlate with enhanced alcohol consummation after ten days. Hence, chronic nicotine exposure increases voluntary ethanol intake and enhances CYP2E1 levels, contributing to the coabuse of these drugs and altering the metabolism of clinical drugs and endogenous substrates [66]. CYP2E1 levels were increased in the frontal cortex and putamen of green monkeys exposed to ethanol alone or in combination with nicotine. The mRNA levels were unaffected by ethanol or nicotine exposure [67]. The brains of alcoholic nonsmokers and alcoholic smokers show greater staining of the granular cells of the dentate gyrus and the pyramidal cells of the CA2 and CA3 hippocampal subregions, as well as of the cerebellar Purkinje cells, compared to nonalcoholic nonsmokers. CYP2E1 induction in the brain by ethanol or nicotine may influence the central effects of ethanol and the development of the nervous tissue pathologies observed in alcoholics and smokers [11]. Nicotine and/or alcohol show induction of CYP2E1 levels in the same brain regions. The coabuse of these drugs may be explained because tobacco smoke constituents increase the metabolic inactivation of ethanol, providing an impetus for increased ethanol consumption. Coaddiction of ethanol and nicotine potentially alters the sensitivity of drugs and toxins in the brain [67]. The modulation of CYP2E1 expression seems to be an adaptive response. Subjects addicted to ethanol and nicotine can respond differently to drugs and endogenous compounds because of this enhanced CYP2E1 expression in the brain [11, 67, 68].

Other exogenous compounds alter CYP2E1 protein and mRNA levels. Nanoparticles, such as titanium oxide (TiO_2_), widely used in toothpastes, sunscreens, and products for cosmetic purpose, accumulate in the brain. Mice exposed to nasal administration of TiO_2 _(10 mg/kg, 90 days) develop oxidative stress and tissue necrosis as well as hippocampal cell apoptosis accompanied by increased expression of genes involved in brain toxicity, including CYP2E1 [69]. Porphyria is an inherited disorder of the heme metabolism that displays neurological symptoms. Porphyrogenic agents include enflurane, isoflurane, and ethanol. Chronic isoflurane anesthesia (1 mL/kg, 10 doses, i.p.) induced CYP2E1 protein expression and acute enflurane anesthesia (2 mL/kg, 1 dose, i.p.) treatment induced CYP2E1 activity in the mitochondrial fraction of mice brains. In the microsomal brain fraction, isoflurane (chronic and acute treatment) diminished CYP2E1 protein levels. These results support an emergent role of CYP2E1 in the pathogenesis of neurological disorders, indicating that CYP2E1 response in the brains of mice could be one of the multiple factors influencing acute porphyria attacks [70]. Brains of streptozotocin-induced diabetic rats, a model reproducing a state of insulin deficiency, mitochondrial CYP2E1 protein, and activity were enhanced in several tissues including the brain. Concomitantly, a marked increase in mitochondrial oxidative stress was observed in brain. Raza et al., 2004, suggested that the induction of mitochondrial CYP2E1 in brain may contribute to the clearance of different compounds, without the presence of exogenous derivatives and induce deleterious effects in the brain [15, 71]. CYP2E1 generates large amounts of ROS that can damage cellular and mitochondrial components, such as mitochondrial DNA and cytochrome c oxidase, enhancing local and cellular oxidative stress [72].

Chronic treatment of phenobarbital anesthesia in African green monkeys induces enhanced CYP2E1 protein levels in the cerebellum and in the putamen [73]. Some xenobiotic compounds, such as pesticides, also modulate CYP2E1 protein levels and activity. Low dose, prenatal exposure to the pesticide lindane produces overexpression of xenobiotic metabolizing enzymes, including CYP2E1, in the brain and liver of postnatal offspring (3 weeks rats) and could be related to behavioral changes observed in these rats [74]. Other pesticides like transfluthrin or D-allethrin pyrethroid vapors induce CYP2E1 and CYP3A2 proteins in the brain. This protein overexpression correlates with an increase in their catalytic activity [75]. Other pesticides such as cypermethrin and propetamphos combinations induce increased CYP2E1 expression and a decrease of glutathione, a major cellular antioxidant, especially in the brain [76]. The antituberculosis drug, isoniazid, induces CYP2E1 expression in primary cerebellar granule neuronal cultures [77].

As in chemical activation, certain circumstances such as a proinflammatory environment or infectious conditions, CYP2E1 are also activated [78]. In vitro studies show that lipopolysaccharide (LPS) treatment in primary cortical glial cultures induced CYP2E1 activity and increased mRNA levels [40, 79]. Using ischemic injury in gerbils and rats, CYP2E1 has been found to be induced in astrocytes, cerebral vessels, and neurons [40, 43, 80, 81]. In diabetic rat models induced with streptozotocin, the consumption of aspartame and insulin treatment increases CYP2E1 activity and protein levels in the brain, without modifying levels and activity in the liver. The induction of CYP2E1 in the brain could have important in situ toxicological effects, given that this CYP isoform is capable of bioactivating various toxic substances and increasing susceptibility to neurotoxic processes [30].

In experimental rat models of hyperlipidemia combined with cerebral ischemia, reperfusion injury increases the protein expression of CYP2E1 combined with enhanced CYP2E1 protein expression and levels of proinflammatory factors. Meanwhile, this study also shows that hyperlipidemia significantly enhances cerebral ischemia/reperfusion- (I/R-) induced transfer of cytochrome c from mitochondria to cytosolic and the protein expressions of Apaf-1 and caspase-3 but also decreases cerebral I/R-induced bcl-2 protein expression. These results reveal that hyperlipidemia exacerbates cerebral I/R-induced injury through the synergistic effect of CYP2E1 induction, which further induces ROS formation, oxidative stress, inflammation, and neuronal apoptosis by the coexistence of hyperlipidemia and cerebral I/R.

Aquaporin-4 participates during the drug metabolism and the detoxification of exogenous substances, aquaporin-4 knockout astrocytes, increased CYP2E1 mRNA expression compared to wild-type astrocytes. CYP2E1 inhibitors protect the cell from damage and the production of ROS induced by 1-methyl-4-phenylpyridinium ions (MPP^+^), LPS, and ethanol in wild-type primary astrocytes [82].

## 5. CYP2E1 Regulation of Expression in the Brain

The induction of CYP2E1 by alcohol appears to be through translational, posttranslational (protein stabilization), and transcriptional mechanisms [83].

CYP2E1 induction involves posttranscriptional stabilization of CYP2E1, unlike other CYP isoform induction processes involving de novo RNA and protein synthesis. The induction of CYP2E1 seems to be regulated at the posttranscriptional or posttranslational levels by the stabilization of mRNA [84] or by protection against the rapid degradation of protein [85] in the liver. The posttranscriptional regulation would be responsible for not only the inducible, but also the constitutive expression of CYP2E1 in liver [86]. Studies in rat and monkey brains demonstrate that CYP2E1 mRNA levels do not increase after ethanol or nicotine treatment, suggesting a nontranscriptional regulation in the brain [44, 67]. Recent works have shown that mRNA CY2E1 expression is modulated by microRNAs, miR-552 [87].

Transcriptional regulation of CYP2E1 has not been extensively examined because its induction in most circumstances has been found to be posttranscriptional. Transcription activation of CYP2E1 has been reported principally during development [83]. Moreover, prenatal exposure to antidepressant drugs such as the serotonin reuptake inhibitor increased the DNA methylation status at the CYP2E1 gene. Furthermore, alteration in birth weight was associated with the neonatal CYP2E1 DNA methylation status [83]. Astrocytes exposed to LPS induce MKKK3 activation, which in turn stimulates a C/EBP and binding element that mediates transcriptional activation of CYP2E1 [79].

We revised in this work, where CYP2E1 is increased in the brain, it may contribute to the clearance of different compounds but also generates ROS without the need for a ligand to produce damage on mitochondria, DNA modification, lipid peroxidation, elevated cytokine production, and even cell death. The alteration of the normal metabolism of endogenous and xenobiotic compounds increases the risks of neurotoxicity by compound bioactivation by environmental chemicals that are metabolized to more toxic derivatives or to procarcinogens by CYP2E1. The regulation of CYP2E1 induction still needs more evidence to be clarified.

## 6. CYP2E1 and Oxidative Stress in Brain

Even though in SNC antioxidant defense has lower expression and it is less efficient. Brain contains nonenzymatic and enzymatic antioxidants system to avoid oxidative damage by ROS. They are vitamins (A, C, and E), low weight molecules (glutathione GSH/GSSG cycle), *β*-carotene, uric acid, alpha lipoic acid, and also enzymes such as GSH-related (GSH-peroxidase, GSH-transferases, thioredoxin, and peroxiredoxin-2), superoxide dismutase, catalase, and peroxiredoxin-1 [88–91].

The activation or enhanced levels of CYP2E1 by different chemicals are sometimes accompanied by the generation of ROS, which may lead to macromolecular damage such as lipid peroxidation and DNA oxidation. Activity of CYP2E1 mainly generates superoxide anion [92] and hydrogen peroxide. In animals with cerebral induced I/R and hyperlipidemia, CYP2E1 induction exacerbates neurological deficit and increases ROS formation, oxidative stress, inflammation, and neurodegeneration [93]. Oral coadministration of vitamin E (200 mg/kg BW) attenuated the neurotoxic effects of deltamethrin (0.6 mg/kg BW), by decreasing oxidative stress, DNA fragmentation, and the expression of CYP2E1, TP53, and COX2 genes. Similarly, the neuroprotection effect has been reported with flavonoids in PC12 cells [94].

**Figure 2:**
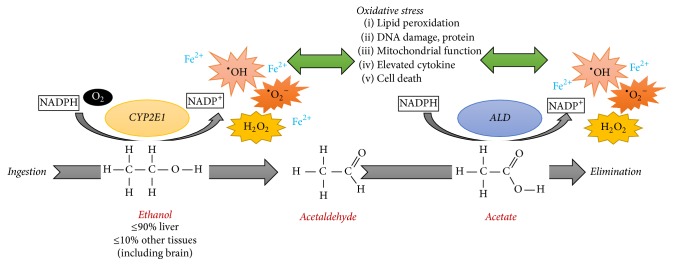
Ethanol oxidation by CYP2E1 results in an increase of ROS and oxidative stress. Ethanol can induce the expression/activity of CYP2E1 resulting in an increase of ROS and cellular damage. Increased ROS levels damage biomolecules such as lipid, protein, DNA, and mitochondria, starting a feedback cycle of ROS production-damage. ALD: aldehyde dehydrogenase; CYP2E1: cytochrome isoform 2E1; ^•^OH: hydroxyl radical; ^•^O_2_^−^: superoxide anion radical; H_2_O_2_: hydrogen peroxide; Fe^2+^: ferrous iron; NADPH: nicotinamide adenine dinucleotide phosphate (oxidized form); NADP^+^: nicotinamide adenine dinucleotide phosphate (reduced form).

Exposure to some active compounds such as ethanol, isoniazid, TiO_2_, and MPP^+^ induces CYP2E1. LPS generates ROS and is accompanied by oxidative stress markers in vivo and in vitro [60, 69, 77, 82, 95]. The brain contains a large amount of phospholipids that are rich in polyunsaturated fatty acids that are liable to peroxidation by ROS, besides the limited regenerative capacity of the brain [53]. CYP2E1 induction leads to increased lipid peroxidation and apoptosis, resulting in increased permeability of the brain blood barrier (BBB) and neurodegeneration, resembling what happens with BBB impairment in alcohol abusers [47]. CYP2E1 shows a higher rate of oxidase activity in purified microsomes compared to other forms of CYP450 [96]. An important substrate of CYP2E1 is molecular oxygen, which displays a high NADPH oxidase activity, with the uncompleted reactions of CYP2E1 leading to the generation of species of free radical [97, 98]. Ethanol metabolism yields to alteration in cellular redox state. In this condition mitochondria exacerbates the production of reactive oxygen and nitrogen species. CYP2E1 activity to ethanol oxidation to acetaldehyde uses H^+^ from NADPH as well as O_2 _resulting in production of large quantities of H_2_O_2_ and superoxide anion radical (^•^O_2_^−^). Depletion of NADPH as well as O_2_ results in the production of large quantities of H_2_O_2_ and the superoxide anion radical (^•^O_2_^−^). Depletion of NADPH can interfere with the respiratory chain: this condition is key to oxidative stress by CYP2E1 activity. Additionally, aldehyde metabolite increases mitochondrial damage that can result in oxygen reduction to ^•^O_2_^−^. CYP2E1 activity can occur in the presence or absence of the CYP2E1 substrate, sensitizing the cells to macromolecular damage and starting a feedback cycle (Figure 2) [99]. CYP2E1 generates ROS such as the radical superoxide anion and hydrogen peroxide in the presence of an iron catalyst and powerful oxidants such as the hydroxyl radical [100]. ROS generated by CYP2E1 may lead lipid peroxidation and its products, such as 4-hydroxynonenal, which binds to DNA, forming highly carcinogenic exocyclic ethane DNA-adducts [101]. A reduced antioxidant environment results in ROS production, DNA oxidation, and cell death. These effects are attenuated by the inhibition of CYP2E1 [77]. An in vitro study of monocytic cell lines demonstrates that the PKC/JNK/SP1 pathway is involved in the induction of CYP2E1 via ROS [95].

CYP2E1 expressing cells were found to be toxic when GSH was depleted by treatment with 1-buthionine sulfoximine (BSO) and CYP2E1 inhibitors prevented the toxicity by the above treatment. HepG2 cells overexpress CYP2E1 (E47 cells) but not the control C34 HepG2 cells, which do not express CYP2E1. E47 cells had a significant 30% increase in the total GSH compared to C34 cells. E47 cells have increased catalase, cytosolic, and microsomal glutathione transferase and heme oxygenase-1 (HO-1) compared to control HepG2 cells, due to activation of their respective genes. This upregulation of antioxidant genes may reflect an adaptive mechanism in removing CYP2E1-derived oxidants [102]. The same working group shows that basal levels of Nrf2 protein and mRNA were higher in the CYP2E1-expressing E47 cells compared to the C34 cells [103]. It has been proposed that Nrf-2 activation is mediated via ROS and free radicals derived from substrates generated by the enhanced activity of CYP2E1 in mice livers [104].

Evidence has demonstrated the role of CYP2E1 over ROS production. Depending on the metabolized substrates and the nature of the compound, CYP2E1 can produce electrophilic compounds that can cause cell toxicity by reacting with cellular macromolecules. As we mentioned, CYP2E1, with O_2_ and NADPH, can produce principally superoxide anions and hydrogen peroxide causing cell damage by macromolecule oxidation.

## 7. CYP2E1 Relationship with Brain Disorders

Clinical studies of Parkinson's disease (PD) show that environmental factors, xenobiotics, including chemicals, pesticides, and volatile solvents are the most suspected causes, all of which are substrates of CYP2E1 [105]. CYP2E1 is present in dopaminergic neurons of the substantia nigra in the same compartment as tyrosine hydroxylase [11, 42, 106, 107]. Also, CYP2E1 mRNA is detected in the basal ganglia and in the substantia nigra [108, 109]. Experimental data show that alcohol reduces the dopamine levels in the midbrain, even if contradictory data is present in the literature and there is an increased oxidative stress in the nigral cells [110, 111]. Nissbrandt et al. in 2001 [112] demonstrated that CYP2E1 activity affects dopaminergic neurotransmission of the substantia nigra, possibly by participating in the metabolism of dopamine. CYP2E1 produces toxic reactive intermediates from endogenous or exogenous substrates, which in turn triggers a chronic impairment of the DA neurons neuronal viability.

It has been shown that CYP2E1 is involved in the 1-methyl-4-phenyl-1,2,3,6-tetrahydropyridine (MPTP) induced mouse model of PD [108, 109]. Acetaldehyde increases the toxic effect of MPTP in striatum [113–115]. In the MPTP mouse model of PD, the inhibition of CYP2E1 with diethylcarbamate increase CYP2E1 toxicity, enhancing DA cell death. Likewise, CYP2E1 null mice did not show any enhanced sensitivity to MPTP or any (1-methyl-4-phenylpyridinium) MPP^+^ accumulation, suggesting a compensatory role of other isozymes that homologue CYP2E1 function [109]. Another report from the same research group states that the lack of CYP2E1 did not increase MPTP toxicity, as they previously reported. In contrast, CYP2E1 mice are weakly sensitive to MPTP-induced brain lesions [116]. Cell cultures from CYP2E1 null mice accumulate more intracellular MPP+ than the cell culture from wild-type mice [109]. Also, MPP^+^ accumulates inside the neurons from Knockout CYP2E1 mesencephalic cultures twice as much as wild-type embryos [105]. This evidence suggests further that CYP2E1 plays a role in MPP^+^ storage and efflux. The accumulation of MPP^+^ in dopaminergic neurons results in the generation of ROS by the mitochondria, including nitric oxide, superoxide anion, hydrogen peroxide, and hydroxyl radicals [117, 118]. MPP+ also stimulates the release of DA [119], and autooxidation of dopamine results in the formation of cytotoxic quinones and highly reactive hydroxyl radicals, generating biomolecular damage [120]. In contrast to this, neuroprotection CYP2E1-regulated mechanism has been observed in a hypoxia model [121].

CYP2E1 brain overexpression has been proposed beneficial in an experimental model of PD [109]. Some compounds related to addictive behaviors, such as smoking and coffee, showed protection against PD [122, 123]. Nicotine subcutaneous injections in rats have been associated with central nicotinic receptor adaption, a pharmaceutic change observed in brain regions of postmortem smokers' brains and hypothesized to be one pathway by which nicotine exerts its behavioral effects such as tolerance [124, 125]. CYP2E1 could exert beneficial effects through an efficient detoxification of neurotoxins related to PD, such as metals and pesticides, decreasing risk factors of PD like oxygen radical production [126]. Furthermore, this evidence supports the notion that the balance between beneficial and neurotoxic effects of CYP enzyme expression might occur as a function of the disease state.

Population studies have demonstrated a possible association between CYP2E1 polymorphisms and some brain disorders, such as PD [127, 128] and the risk of glioma [129] and pain implications [130]. CYP2E1 gene methylation and increased CYP2E1 mRNA are found in PD patient's brains [131]. These data suggest that epigenetic CYP2E1 alterations may facilitate the degenerative process through the metabolism of such xenobiotics and represent the genetic susceptibility to the disease [105]. These gene of CYP2E1 can influence the body's ability to interact with the detoxificant or the bioactivation of multiple chemical substrates. Kumsta et al., 2016, reported methylation changes in the promoter-regulatory region of the cytochrome P450 2E1 gene in children with clinical markers of impaired social cognition [132].

The overexpression of CYP2E1 has been described in the hippocampal region of postmortem patients with drug-resistant epilepsy and in hippocampal cultures. It has also been described in mice with status epilepticus exposed to the antiepileptic drug phenytoin [28]. In rodents exposed to ethanol, it has been reported that cerebral CYP2E1 activity correlates with locomotor activity, which could suggest that metabolic acetaldehyde is a mediator of some ethanol-regulated pharmacological effects [133]. CYP2E1 could influence sensitivity to ethanol in the SNC [134]. Acetaldehyde in the hippocampus affects synaptic transmission [135].

Acetaminophen causes apoptosis and DNA fragmentation through CYP2E1 mediated JNK activation in C6 glioma cells [136]. Unexpectedly, the researchers found that acetaminophen reduced p53 proapoptotic protein and the necessary doses of acetaminophen that induced cell death, despite the stimulation of p53 phosphorylation in C6 glioma cells through CYP2E1 [137].

CYP2E1 exerts many functions in the metabolism of different endogenous or exogenous compounds, including bioactivation, degradation, flux, and storage. Alterations in these functions of CYP2E1 are related to different brain disorders. The genetic variant or epigenetic changes to CYP2E1 make it more susceptible to an efficient metabolism but, conversely, more vulnerable to bioactive compounds formed through CYP2E1 activity and has been related to different brain disorders (Table 2).

## 8. Ethanol Oxidation in Behavioral Alterations and CYP2E1

Three enzymes are responsible for oxidizing ethanol to acetaldehyde: aldehyde dehydrogenase (ADH), catalase, and CYP2E1 [138]. Catalase is the primary ethanol metabolizing enzyme in the brain. Studies have revealed it to be responsible for approximately 50% of ethanol metabolism occurring in the brain [139]. In neurons and monocytes/macrophages, ADH is present at very low levels in these cells; thus the involvement of CYP2E1 is greater than ADH [85]. Animal models using genetic knockout of CYP2E1 and/or catalase indicate that CYP2E1 is responsible for ≈20% of ethanol metabolism in the brain. Catalase and CYP2E1 inhibitors diminish the accumulation of the ethanol derived acetaldehyde and acetate in brain homogenates. Inhibitors of ADH decrease the acetate but not the acetaldehyde [139]. The absence of CYP2E1 in brain homogenates of CYP2E1 knockout mice was not affected by acetaldehyde levels, bringing into question the importance of this enzyme in ethanol oxidation [138]. The mRNA P450s from CYP2E1 has been observed in human amygdala and prefrontal cortex of alcoholics and smokers, areas associated with addictive behaviors [140], suggesting that this expression may be altered by alcohol and tobacco and influenced by the normal metabolism of exogenous and endogenous chemicals by CYP2E1.

Different ethanol-associated behavior alterations are attributed to acetaldehyde toxicity, such as euphoria, anxiolytic, hypnotic, amnesiac, and aggression, as well as reinforcement or aversion to voluntary ethanol consumption (preference) [141–144]. Physiological alterations caused by acetaldehyde in glia include alterations in cell function, growth, and differentiation [145]. Studies contradict the theory that acetaldehyde contributes to ethanol's hypnotic effect, shown in anticatalasemic mice or CYP2E1 null or catalase/CYP2E1 dual deficient mice. In this sense, a decrease in blood acetaldehyde levels was accompanied by an increase in ethanol-induced sleep time, especially with high doses of ethanol [138]. In 2009 Correa et al. showed that, with the lack of the CYP2E1 enzyme, the consequence was an increase of catalase levels, as a compensatory detoxifying metabolism. The lack of CYP2E1 has an impact over ethanol-induced sensitization and on voluntary ethanol preference in knockout CYP2E1 mice after repeated intermittent alcohol intake showed a reduction in preference for ethanol intake compared with wild-type mice [146]. These results suggest that the role of CYP2E1 in ethanol oxidation of acetaldehyde and their behavioral alterations in the brain needs to be clarified.

## 9. CYP2E1 in Inflammatory and Autophagy Processes

The metabolism of ethanol induces brain damage and neurodegeneration by triggering inflammatory processes in glial cells through the activation of Toll-like receptor 4 (TLR4) signaling [147]. Chronic ethanol consumption impairs proteolytic pathways in mouse brains and the immune response mediated by TLR4 receptors participates in these dysfunctions [147]. Recent studies have shown that autophagy serves as a protective mechanism against ethanol-induced injury. Autophagy was found to be protective against CYP2E1-dependent toxicity in vitro in hepatic HepG2 cells that express CYP2E1 and in vivo in an acute alcohol/CYPE1-dependent liver injury model [148]. MTOR pathway integrates cellular signals and mediated autophagy activation is also a neuroprotective response that alleviates ethanol toxicity [149, 150].

An immature brain is more susceptible to ethanol neurotoxicity. Fetal alcohol spectrum disorder (FASD) results from ethanol exposure to the developing fetus and is the leading cause of mental retardation. FASD is associated with a broad range of neurobehavioral deficits which may be mediated by ethanol-induced neurodegeneration in the developing brain. The vulnerability of the immature brain to ethanol shows a high expression of proapoptotic proteins and responsive stress system deficiency, such as unfolded protein response and autophagy [151].

## 10. Concluding Remarks

In summary, this paper shows differential expression of CYP2E1 in brain regions. The activity of CYP2E1 is similar to the metabolism of endogenous and xenobiotic compounds. CYP2E1 is a highly conserved enzyme related to a diversity of effects in the mammalian brain. The differential expression of CYP2E1 in the brain suggests that some regions are more susceptible to an efficient detoxification or to cell damage, principally through the generation of oxidative stress, as a result of different molecular metabolisms of CYP2E1. CYP2E1 expression has been involved on behavioral and locomotor activity or in the neurophysiology in different toxicological animal models and human diseases. In this work, we have revised the modulation of CYP2E1 by different xenobiotics or pathological situations (Figure 1), demonstrating not only the different brain targets of CYP2E1, but also the mechanisms throughout the physiological damage in the brain. Further studies are required to clarify how the mechanisms of CYP2E1 induction are regulated in the brain and how environmental and pharmacological factors induce it, in addition to cell or region vulnerability in the brain. It is necessary to know if the polymorphisms, expression, and activity of CYP2E1 are related to either ethanol or nicotine preference or dependence and its effects on the susceptibility in the human population. In this regard, it is important to explain the role of CYP2E1 in different regions of the CNS and its contribution to addictive behaviors and in neurodegenerative processes.

## Figures and Tables

**Table 1:** CYP2E1 brain distribution in rat, human, and monkey brains.

Species	Region	CYP2E1	Detection method	Reference
Rat	Olfactory lobe	mRNA	RT-PCR	[37]
			in situ hybridization	[45]
		Protein	Western blot	[39, 40, 43]
		Activity	HPLC chlorzoxazone method	[45, 152]
	Cortex	mRNA	In situ hybridization	[45]
		Protein	Immunohistochemistry	[11, 43]
		Activity	HPLC chlorzoxazone method	[45, 152]
	Hippocampus	mRNA	In situ hybridization	[41]
		Protein	Immunohistochemistry	[11, 38]
		Activity	HPLC chlorzoxazone method	[40, 41]
	Cerebellum	mRNA	In situ hybridization	[45]
		Protein	Immunohistochemistry	[11, 43]
		Activity	HPLC chlorzoxazone method	[45, 152]
	Striatum	mRNA	In situ hybridization	[45]
		Activity	HPLC chlorzoxazone method	[45, 152]
	Thalamus	mRNA	In situ hybridization	[45]

Human	Cortex	Protein	Immunohistochemistry	[11]
		mRNA	In situ hybridization, PCR	[41, 48]
		Activity	HPLC chlorzoxazone method	[41]
	Cerebellum	Protein	Immunohistochemistry	[11]
		mRNA	In situ hybridization, PCR	[41, 48]
		Activity	HPLC chlorzoxazone method	[41]
	Hippocampus	Protein	Immunohistochemistry	[11]
		mRNA	In situ hybridization	[41]
		Activity	HPLC chlorzoxazone method	[41]
	Eye	mRNA	RT-PCR	[50]
	Pons	mRNA	PCR	[48]
		Activity	HPLC chlorzoxazone method	[41]
	Substantia nigra	mRNA	PCR	[48]
	Striatum	Activity	HPLC chlorzoxazone method	[41]
	Thalamus	Activity	HPLC chlorzoxazone method	[41]

Human prenatal brain		Protein	Western blot	[49]
		mRNA	RT-PCR	[49]
		Activity	HPLC nitrophenol method	[49]

Monkey	Cerebellum	Protein	Immunohistochemistry	[44]
	Cortex	Protein	Immunohistochemistry	[44]
	Hippocampus	Protein	Immunohistochemistry	[44]
	Substantia nigra	Protein	Immunohistochemistry	[44]

**Table 2:** CYP2E1 polymorphisms related to brain pathologies.

CYP2E1 polymorphism	Brain pathologies	Inductor agent	Reference
C1/C2	Polyneuropathy	Isoniazid	[153]
Rs6413419 G	Alcohol dependence	Alcohol	[154]
CYP2E1^*∗*^1D	Motor neuron disease		[140]
CYP2E1 RsaI	Glioma		[129]
